# Impact of three‐dimensional prostate models during robot‐assisted radical prostatectomy on surgical margins and functional outcomes

**DOI:** 10.1111/bju.16850

**Published:** 2025-07-13

**Authors:** Nawal Khan, Davide Prezzi, Nicholas Raison, Andrew Shepherd, Michela Antonelli, Nick Byrne, Maia Heath, Christopher Bunton, Carlo Seneci, Eoin Hyde, Andres Diaz‐Pinto, Findlay Macaskill, Benjamin Challacombe, Jonathan Noel, Christian Brown, Ata Jaffer, Paul Cathcart, Margherita Ciabattini, Armando Stabile, Alberto Briganti, Giorgio Gandaglia, Francesco Montorsi, Sebastien Ourselin, Prokar Dasgupta, Alejandro Granados

**Affiliations:** ^1^ School of Biomedical Engineering & Imaging Sciences King's College London London UK; ^2^ Department of Urology Guy's Hospital London UK; ^3^ Medical Physics and Clinical Engineering Department Guy's and St Thomas' NHS Foundation Trust London UK; ^4^ Innersight Labs London UK; ^5^ Unit of Urology San Raffaele Hospital Milan Italy; ^6^ NVIDIA Santa Clara CA USA

**Keywords:** prostate cancer, robot‐assisted radical prostatectomy, 3D printed models, 3D virtual models, positive surgical margins, incontinence, potency, sexual function

## Abstract

**Background:**

Robot‐assisted radical prostatectomy (RARP) is the standard surgical procedure for the treatment of prostate cancer. RARP requires a trade‐off between performing a wider resection in order to reduce the risk of positive surgical margins (PSMs) and performing minimal resection of the nerve bundles that determine functional outcomes, such as incontinence and potency, which affect patients’ quality of life. In order to achieve favourable outcomes, a precise understanding of the three‐dimensional (3D) anatomy of the prostate, nerve bundles and tumour lesion is needed.

**Study Design:**

This is the protocol for a single‐centre feasibility study including a prospective two‐arm interventional group (a 3D virtual and a 3D printed prostate model), and a prospective control group.

**Endpoints:**

The primary endpoint will be PSM status and the secondary endpoint will be functional outcomes, including incontinence and sexual function.

**Patients and Methods:**

The study will consist of a total of 270 patients: 54 patients will be included in each of the interventional groups (3D virtual, 3D printed models), 54 in the retrospective control group and 108 in the prospective control group. Automated segmentation of prostate gland and lesions will be conducted on multiparametric magnetic resonance imaging (mpMRI) using ‘AutoProstate’ and ‘AutoLesion’ deep learning approaches, while manual annotation of the neurovascular bundles, urethra and external sphincter will be conducted on mpMRI by a radiologist. This will result in masks that will be post‐processed to generate 3D printed/virtual models. Patients will be allocated to either interventional arm and the surgeon will be given either a 3D printed or a 3D virtual model at the start of the RARP procedure. At the 6‐week follow‐up, the surgeon will meet with the patient to present PSM status and capture functional outcomes from the patient via questionnaires. We will capture these measures as endpoints for analysis. These questionnaires will be re‐administered at 3, 6 and 12 months postoperatively.

**Abbreviations**
2D: two‐dimensional
3D: three‐dimensional
mpMRI: multiparametric MRI
NVB: neurovascular bundle
PSM: positive surgical margin
RARP: robot‐assisted radical prostatectomy

## Background

Prostate cancer is the most common cancer in the United Kingdom, affecting one in eight men and causing the death of over 11 500 men per year [1]. Specifically, in 2020, the worldwide prostate cancer incidence, mortality and 5‐year prevalence were 14.1%, 6.8% and 20%, respectively [2].

Robot‐assisted radical prostatectomy (RARP) is the standard surgical approach to treat prostate cancer. Cancer extending beyond the prostate capsule increases the risk of positive surgical margins (PSMs), which are independent predictors of prostate cancer and biochemical recurrence after RARP [3]. RARP requires a trade‐off between performing a wider resection in order to achieve low PSM rates (11%–37% on average [4, 5]), and performing a narrower resection to preserve structures that determine functional outcomes, such as incontinence and potency, which affect patients’ quality of life [6]. Urinary incontinence remains one of the most troublesome postoperative complications, with aetiology that is complex, multifactorial, and affected by surgical technique, surgeon's skill and patient characteristics [7]. Erectile dysfunction is of particular concern after RARP, with prevalence varying from 14% to 90% [8].

Multiparametric MRI (mpMRI) is increasingly used to support the diagnosis, tumour localisation, and management of prostate cancer through three‐dimensional (3D) T2‐weighted imaging, diffusion‐weighted imaging with apparent diffusion coefficient, and dynamic contrast‐enhanced sequences [9]. mpMRI is also used by surgeons for surgical planning since a good understanding of the anatomy is essential to reduce the risk of complications. However, surgeons are expected to mentally reconstruct conventional two‐dimensional (2D) mpMRI into a 3D representation that matches the anatomy as seen through an endoscope during RARP [10]. Precise knowledge of the 3D location of the tumour and its relationship to the prostate capsule and neurovascular bundles (NVBs) can inform the surgical management and nerve‐sparing technique, whilst reducing PSMs [11, 12]. Although studies have shown that mpMRI induces surgeons to change their surgical plan, reducing PSMs [13], it is difficult for surgeons to imagine intra‐operatively the precise location of tumours entirely from preoperative multiparametric medical images, a process that becomes less challenging with experience.

Some methods have been proposed to support the understanding of a patient's anatomy during surgery. 3D surgical navigation models have been displayed next to the surgeon console [12], overlaid on endoscopic videos using elastic‐based augmented reality [11], and made available to surgeons as 3D printed models during surgery [14]. Despite the variability reported in these studies, evidence from a recent literature review and meta‐analysis [15] suggests a trend towards a reduction in PSMs [11, 12, 14], with no clear evidence of the impact on functional postoperative outcomes. That review and meta‐analysis of the potential benefits of 3D models highlighted limitations in current studies, including a lack of comparison between 3D printed and virtual models, lack of description and justification for the methodology chosen, and studies contradicting each other. Moreover, the role of 3D virtual and 3D printed models in surgery is yet to be defined as there are many covariates that have an effect on outcomes, such as surgeon's expertise, location of cancer lesions, number of anatomical structures to be included, approaches for the creation of 3D models, among others.

### Study Design

**Fig. 1:**
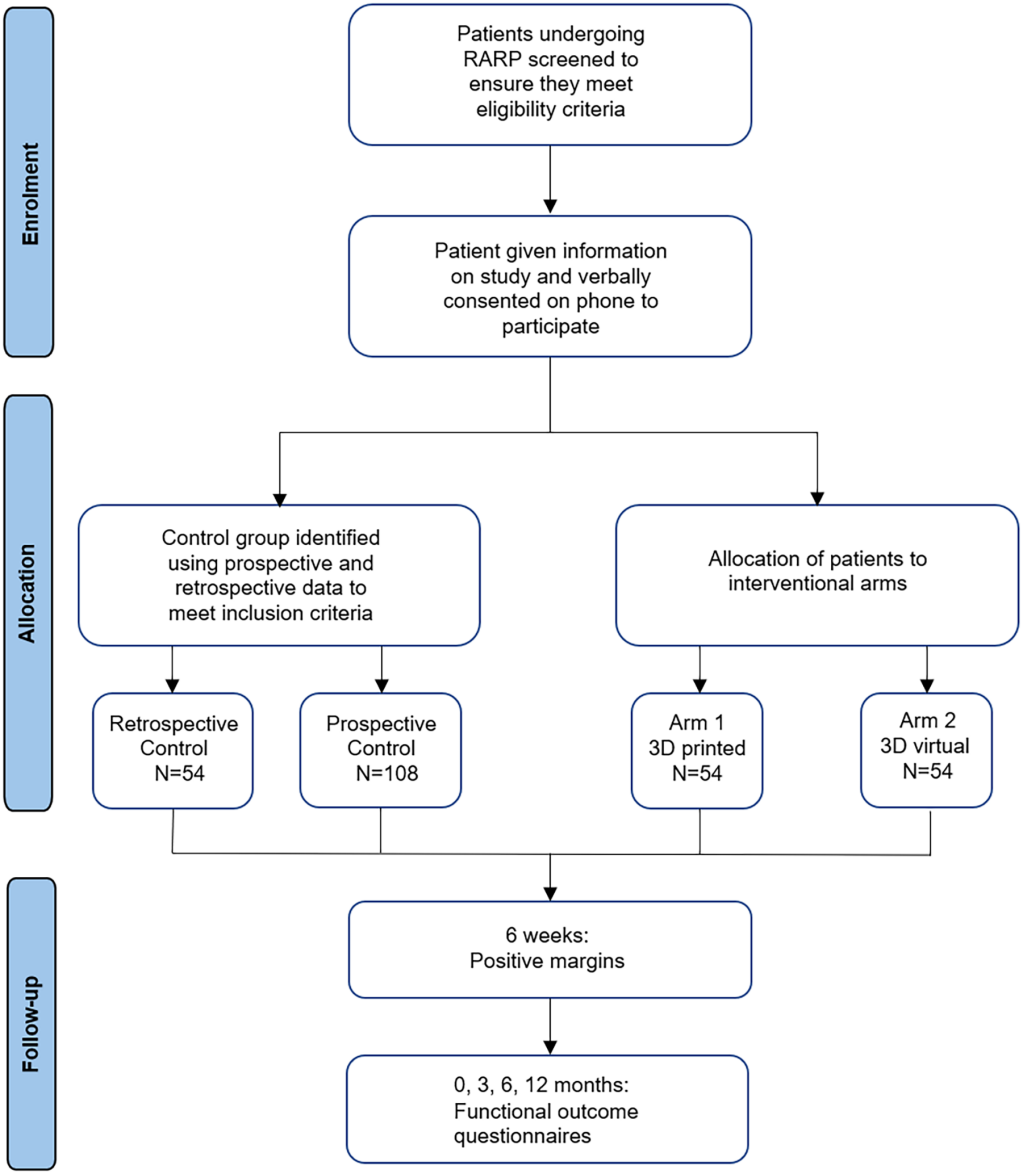
Flow of participants and execution of our feasibility study. RARP, robot‐assisted radical prostatectomy.

This is the design for a single‐centre parallel feasibility study consisting of two prospective intervention arms (a 3D printed model and a 3D virtual model), and a retrospective and a prospective control group (Fig. 1). Although this study will be non‐blinded for surgeons as they will be operating on patients using either of the models, it will be blinded for analysis. Note that this will not be a randomised study.

The aim of this study is to measure the effect of 3D models on the reduction of PSM rates and on functional outcomes after RARP. Our main hypothesis is that there will be a reduction of positive resection margins (PSMs) and improvement of functional outcomes for patients undergoing RARP when surgeons are presented with a 3D printed or 3D virtual patient‐specific prostate model during surgery. Specifically, we hypothesise that 3D models reinforce the mental reconstruction of the anatomy that surgeons acquire via conventional 2D mpMRI whilst assisting surgeons in matching the precise location of the anatomy and lesions with respect to the endoscopic view they observe during RARP [10]. 3D models could inform the surgical management and nerve‐sparing technique, whilst avoiding increases in PSM rates. Studying both 3D virtual and 3D printed models will allow us to understand whether tactile information is important. We highlight the importance of recruiting surgeons across different levels of expertise to reduce bias and avoid having expertise as a confounder for the impact of 3D models on the reduction of PSMs. Therefore, we will stratify our results based on each surgeon's number of years of experience.

We will also report measures related to the deployment of this feasibility study in anticipation of a subsequent larger randomised controlled trial. Measures include patient recruitment rate, and percentage of cases that led to successful model deployment in the operating theatre.

### Endpoints

The primary study outcome is PSM rates measured after specimen analysis. Secondary outcomes include incontinence rates (pad weights, electronic Patient‐Reported Outcome Measures), erectile dysfunction (International Index of Erectile Function score, and Erectile Hardness Score), and International Consultation on Incontinence Questionnaire‐Urinary Incontinence (ICIQ‐UI) score captured at 3 months after surgery. Other secondary outcomes are related to our pipeline deployment metrics, including patient recruitment rate and percentage of cases that led to successful model deployment in the theatre.

### Eligibility Criteria

Men will be included in the study provided they meet the following criteria:

1. T2–T3a prostate cancer lesions located on the capsule and near NVBs;
2. Gleason score ≥3 + 4; and
3. Eligibility for RARP after assessment of mpMRI during multidisciplinary team meetings according to local guidelines.

If any of the following criteria are met, the subject must not be included in the study:

1. Prior treatment for prostate cancer;
2. Pre‐existing erectile dysfunction;
3. Pre‐existing urinary incontinence;
4. mpMRI not possible, or assignment to other research studies where functional outcomes are likely to change; or
5. Presence of anterior tumour lesions of the prostate and those confined to the prostate.

## Methods

### Interventions

Patients diagnosed with prostate cancer will undergo discussion at the weekly multidisciplinary team meeting at Guy's Hospital (London, UK) to decide management options. These patients will then be followed up in the clinic to determine those who opted for RARP. A list of those scheduled for RARP in the upcoming months will be collected. Patients will fill out questionnaires in the clinic regarding baseline functional information preoperatively related to potency and continence and these data will be stored on REDCap (Research Electronic Data Capture software). The hospital electronic health record will be used to collect data on patient demographics, staging, Gleason score, prior treatment for prostate cancer, potency and incontinence to eliminate patients not meeting our inclusion criteria.

Patients will then be contacted via telephone and informed about the study. They will be given written information via e‐mail or in person preoperatively on: (i) the aims of the study; (ii) the patient information materials available; and (iii) contact details of chief investigator.

Patients can consent verbally to participate in the study via telephone but can opt to withdraw any time before the surgery. They will then be given a written consent form to sign before their surgery, which will include permission to use their data. Pseudo‐anonymisation will be carried out by a Clinical Research Fellow to allow data processing and analysis by Data Scientists.

Once patients have consented verbally, mpMRI will be obtained and anonymised for extraction of anatomical structures. Histology data will also be collected from previous prostate biopsy to help better identify the exact location and size of lesions. These will then be reviewed by the radiologist to guarantee the patient is eligible given our inclusion criteria.

#### Segmentation of Anatomy on mpMRI

**Fig. 2:**
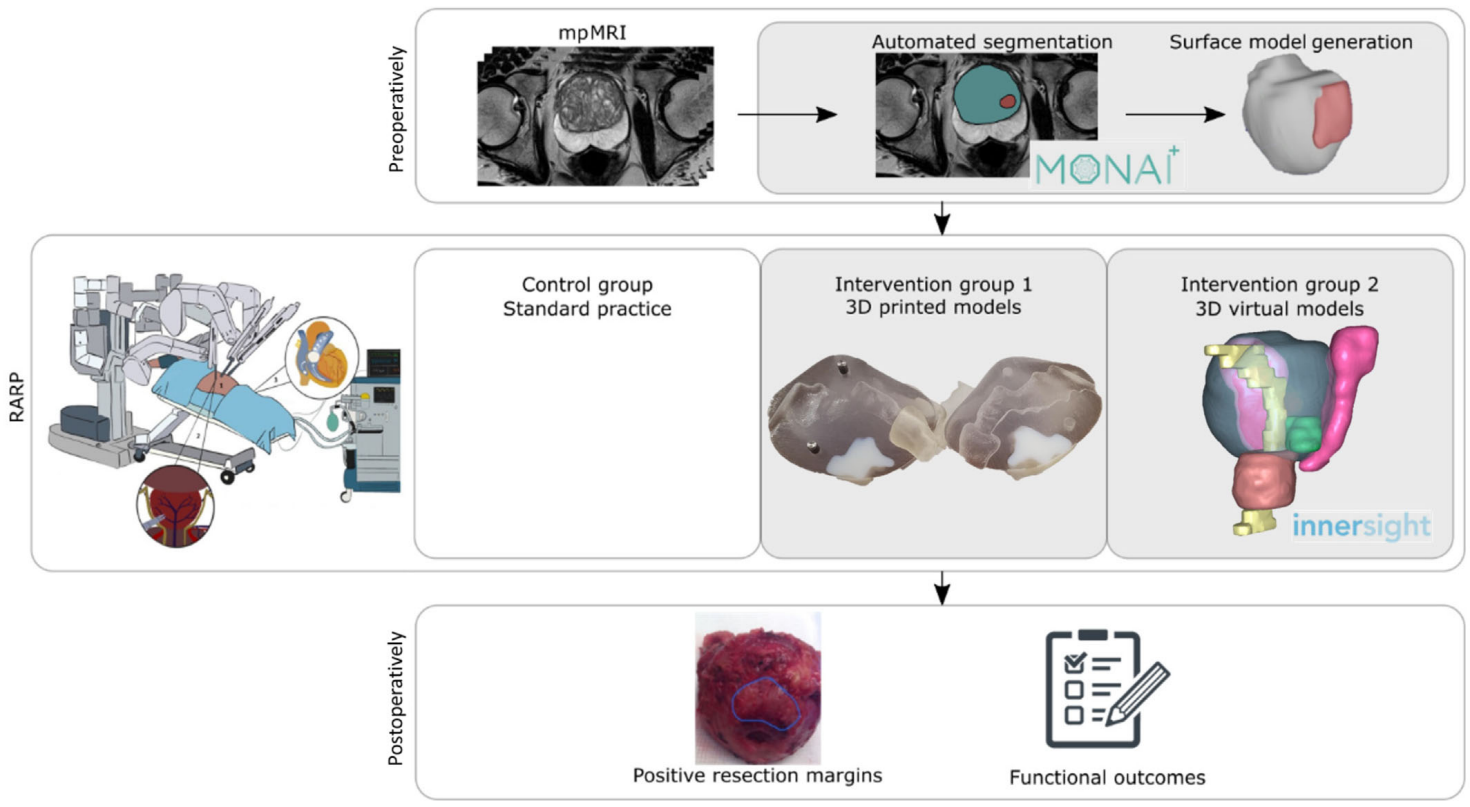
Two‐arm parallel study. Top: muliparametric MRI (mpMRI) is used to construct three‐dimensional (3D) printed or 3D virtual patient‐specific prostate anatomical models preoperatively. Middle: a prospective control cohort and two prospective intervention arms, in which 3D printed or 3D virtual models are given to surgeons during robot‐assisted radical prostatectomy (RARP). Bottom: collection of postoperative surgical outcomes including PSMs and functional outcomes related to erectile dysfunction and urinary incontinence.

**Fig. 3:**
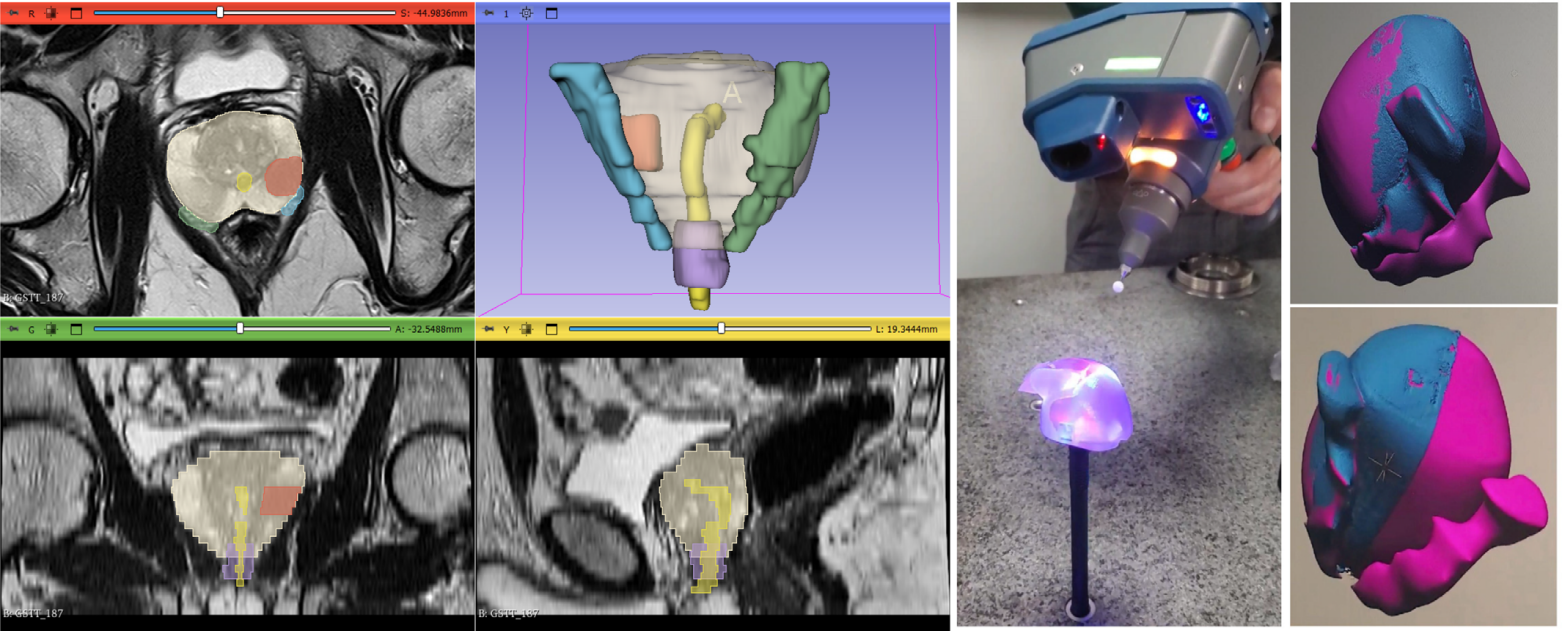
Left: segmentation of patient‐specific prostate anatomy on multiparametric MRI (mpMRI) to extract annotations of the prostate gland (shown in translucent white), tumour lesions (shown in red), external sphincter (shown in purple), urethra (shown in yellow), and neurovascular bundles (shown in blue and green). Right: annotations resulting from segmentations are three‐dimensional (3D) printed and scanned using a coordinate measuring machine that captures a 3D representation of the physical model. The overlapping of annotations (in pink) and scanned 3D representation (in blue) validates that 3D printed models and 3D virtual models fully correspond to each other.

The radiologist will delineate a total of five different prostate‐related anatomical regions on mpMRI using ‘3D Slicer’, a process commonly referred to as image segmentation. First, automated segmentation of the prostate gland will be carried out using ‘AutoProstate’ in MONAI Labels on axial T2‐weighted images [16, 17]. Typical parameters of acquisition of T2‐weighted imaging include slice thickness = 3.5 mm, interslice gap = 3.5 mm, and field of view = 180 mm * 180 mm. The results of prostate segmentation will be validated by the radiologist, and, if necessary, manually corrected. Then, manual segmentation of cancer lesions will be performed on T2‐weighted, apparent diffusion coefficient, and high b‐value diffusion weighted image sequences supported by histological data and patient records. Last, the external sphincter, urethra, and NVBs will be manually segmented on T2‐weighted images (Fig. 2 top and Fig. 3 left).

#### Surface Model Generation

Labels resulting from segmentation will be processed through a pipeline specifically developed for our study. This pipeline will create labels either for 3D virtual models or for 3D printing.

Patients will be assigned to one of the intervention groups based on the resources available to produce 3D printed or 3D virtual models in a timely manner before surgery (Fig. 2). Specifically, the allocation to an intervention arm will be carried out based on the time that the anonymised data are received by the engineering team and the remaining days before surgery to allow the generation of 3D models. Note that virtual models are faster to generate compared to printed models.

#### Control Group

Patients will be prospectively assigned to a control arm according to our inclusion/exclusion criteria. We aim to include cases following 1:2 propensity‐score matching with respect to intervention arms. These cases will be pseudo‐anonymised and data collected will include patient demographics, mpMRI, PSM status, and functional outcomes. For comprehensiveness, we also aim to include retrospective cases observed over the same period of time as the execution of our feasibility study, according to our inclusion/exclusion criteria. This prospective vs retrospective analysis will allow us to measure for any possible bias and validate whether the intervention arms have impact in comparison with historical data. A search for retrospective cases will be conducted over the same period of time as the execution of our feasibility study to reflect similar practice. However, given that bias might be introduced due to the nature of a retrospective arm, we will additionally include cases as part of a prospective control arm during the execution of our study, but only if there are enough cases after interventional arm assignments.

#### Three‐dimensional Printed Intervention Arm

Patients will be prospectively recruited to obtain 3D printed models to surgeons at the start of RARP. These models will be printed by Guy's and St Thomas' Hospitals 3D printing facilities, using an Object500 Connex1 polyjet 3D printer (Stratasys, USA). As part of the design of this protocol, we have developed a bespoke additive manufacturing workflow to meet the requirements of our patient‐specific 3D printing task. The aims of our workflow are: (i) to maximise the clarity with which tumour lesions can be discriminated from healthy glandular tissues, and (ii) to minimise geometrical deviation from the virtual model obtained from segmentation on mpMRI. Our workflow meets these ambitions by leveraging multiple materials (with visually contrasting properties) and multi‐part assembly (to eliminate the need for abrasive post‐processing). The geometrical accuracy of the following patient‐specific 3D printing specification was validated using a coordinate measuring machine (Faro, USA): disagreement between 3D printed and virtual surfaces (see Fig. 3, right panel).

We will print the healthy glandular tissue using a transparent photopolymer (VeroClear FullCure810), with an optically contrasting, opaque, white photopolymer (VeroWhitePlus FullCure835) to identify lesions. Furthermore, we have chosen to print the models in two approximately hemispherical sections (see Fig. 2 middle), the ‘overhang‐free’, monotonic geometry of which minimises the supporting material required. This is beneficial, eliminating the need for the abrasive polishing that risks undue deviation from the intended geometry, and that would otherwise be required to remove vestiges of supporting material to ensure gland transparency. To discriminate the NVBs and external sphincter from the remaining anatomy, we will use a distinct, soft photopolymer (Agilus30 Clear FLX935). The urethra will be demonstrated as a hollow passage through the gland. To ensure models can be traced to specific patients, the pseudo‐anonymised case identifier will be 3D printed (as a recessed label) into the surface of the gland, taking an inconsequential location remote from disease. Following printing, the two hemispheres will be combined using metallic pins, sized to ensure a reversible push‐fit. The sphincter will be held in place using an epoxy adhesive.

The design chosen for this study is the result of an iterative process resulting in 3D models printed in two halves that are easily put together with the aid of two metallic pins to allow the visualisation of urethra and cancer lesions. A pseudo‐anonymised code is automatically engraved into the prostate. The 3D models are printed in three different materials: prostate gland in translucent (VeroClear FullCure810), cancer lesions in white colour (VeroWhitePlus FullCure835), and external sphincter and NVBs in soft material (Agilus30 Clear FLX935), with the urethra showing as hollow.

#### Three‐dimensional Virtual Intervention Arm

Patients will be prospectively recruited to provide 3D virtual models to surgeons at the start of RARP via a laptop (Windows 10 Dell Latitude 3520). Pseudo‐anonymised T2‐weighted images and labels will be uploaded to Innersight Labs, a CE‐marked surgical planning tool, to visualise the prostate anatomical regions in 3D in theatre.

During RARP, a patient‐specific 3D printed or 3D virtual model will be available to the surgeon, who will be able to manipulate it at any time as a reference for the anatomy seen in the endoscopic video during robot‐assisted surgery. Videos currently recorded for RARP as standard practice will be used for analysis of the phases and actions performed during surgery to understand the relationship of the 3D printed/virtual model.

We will ask surgeons to complete a questionnaire after surgery to capture their perspectives in relation to the 3D models made available to them.

### Sample Size Determination

This study will be conducted in a total of 270 patients: a prospective control group (*N* = 108), a retrospective control group (*N* = 54), a prospective 3D printed intervention arm (*N* = 54), and a prospective 3D virtual intervention arm (*N* = 54).

The sample size (*N* = 270) was computed based on a two‐arm study at 0.05 significance level in combination with Dunnett's correction, 0.75 power size, 0.25 interesting treatment effect, and equal standard deviations of 0.5 across arms for a normally distributed primary outcome using a ‘multiarm’ web tool (https://mjgrayling.shinyapps.io/multiarm) [18]. Based on our eligibility criteria controlling for patients with pT2 and pT3 lesions, we hypothesise that we will observe a PSM rate of approximately 20% in our control group, as evidenced from previous studies [15, 19].

### Methods of Data Collection

This will be a single‐centre study performed in the Urology Department of Guy's Hospital, London, UK. Specimen analysis will be carried out on the whole prostate gland removed from the patient during RARP to evaluate margin status and estimate PSMs. There will be no deviation from normal clinical practice.

At the 6‐week follow‐up, the surgeon will meet with the patient to present PSM status and capture functional outcomes from the patient via questionnaires. We will capture these measures as endpoints for analysis. These questionnaires will be re‐administered at 3, 6 and 12 months postoperatively.

### Data Analysis

For analysis, we will report contingency tables, whereby the primary outcomes will be treated as categorical variables. We will use Pearson's chi‐squared probability distribution to compare the three groups (two interventional and one control), whereby both independent (treatment: 3D virtual or 3D printed models) and dependent (PSMs) variables are categorical. Our null hypothesis is that there is no difference among these three groups. We will conduct analysis at the significance level of *P* = 0.05. We will report mean, variability and CIs for the control group and intervention arms of PSMs and functional outcomes. When analysing continuous variables resulting from questionnaires related to functional outcomes, we will conduct parametric (one‐way anova) and non‐parametric (Kruskal–Wallis test) tests depending on the tests of normality, and run an ancova, while adjusting for multiple comparisons (e.g. Bonferroni) and including pairwise comparisons (independent *t*‐tests).

## Discussion

The identification of number and size of prostate cancer lesions prior to RARP has been limited to the 2D view of mpMRI scans and biopsy results. Surgeons are expected to mentally construct and visualise the exact location and dimensions of the lesions in 3D format prior to surgery [10]. The aim of prostate cancer surgery is to preserve maximum nerve function whilst preventing PSMs in order to achieve good functional outcomes. To accomplish this, a better understanding of prostate cancer lesions and the surrounding anatomy is needed [14]. 3D modelling has the potential to enhance surgical planning by providing surgeons with an intra‐operative navigation tool to better appreciate the nature of lesions [11].

A few studies have been conducted using either 3D virtual models or 3D printed models for RARP. Despite varying results, a number have shown a decrease in PSM rates irrespective of the model used; however, only one showed a statistically significant improvement [15]. That study, by Checcucci et al. [20], involved a total of 800 patients, with 3D virtual models created for 640 of these patients. The authors showed a PSM rate of 25% in the 3D virtual model group vs 33% in the control group [20]. To date, most studies have not used inclusion criteria that involve type of lesion, location of lesion, tumour stage or Gleason score [10, 11, 20, 21, 22, 23]. Because a lesion location that is close to the capsule or NVBs can make a significant difference to how conservatively the surgeon approaches the NVBs and the likelihood of PSMs, we have decided to exclude anterior lesions and lesions located far from the capsule to assess the true impact of the models. In addition, most studies have looked at either 3D virtual or 3D printed models [15]. In order for us to assess which is more effective in a similar cohort of patients at a single centre, we have chosen to compare both. This will help us further decide which type of model to use for a randomised controlled trial in the future.

Despite evidence showing a reduction of PSMs, comparatively few data are available showing the impact of 3D prostate models on functional outcomes, with a recent meta‐analysis showing no clear difference in postoperative and functional outcomes in the intervention groups [15]. For this reason, we will follow up patients at regular intervals for up to a year to assess the effects on incontinence and potency.

The surgical lead for this proposal has run an Idea, Development, Exploration, Assessment, Long‐term follow‐up (IDEAL) Phase 2a study on the use of patient‐specific 3D printing in RARP in a cohort of 10 patients with intermediate and high risk of prostate cancer (T3 stages). Despite the small sample size, the outcomes of this study were crucial: PSM rates were reduced from ~35% to 10% [14]. In this study, improvement in tactile appreciation of the size, number and location of the lesions via 3D printing may have proved useful.

In all the work mentioned above, manual delineation of organs on medical images is required, a process known as medical image segmentation. Prostate segmentation is typically performed manually on preoperative T2‐weighted MRI to produce a 3D anatomical shape of the prostate gland, whereas prostate cancer lesion segmentation is conducted manually on mpMRI to generate 3D representations of the location of cancer lesions. Both tasks are time‐consuming and require substantial radiological expertise. Moreover, manual segmentation is not scalable and is prone to human error. Therefore, a reliable automated approach for prostate and cancer lesion segmentation is crucial to overcome some of the limitations of translating 3D virtual models and 3D printed models into clinical practice and into the operating theatre.

Our group has recently made substantial progress towards the automated segmentation of the prostate gland and cancer lesions, developing technologies that are timely and innovative for this proposal. In particular, our group has designed MONAI Labels, a labelling and learning tool that helps researchers and clinicians rapidly build artificial intelligence models to create segmentation datasets, for use in a study currently under review [24].

## Participant Discontinuation and Withdrawal Criteria

Patients can choose to completely withdraw from the study before surgery. Once patients sign the written consent form prior to surgery, data will be collected. However, one foreseeable circumstance is a patient dying before or just after surgery, which will make data collection impossible. If a patient decides to drop out of the study after providing consent, they will be withdrawn from our study, but their data will still be used to report metrics related to the implementation of our study.

Patients who withdraw from the study will be replaced by other patients following covariate assignment to avoid bias within a cohort and to guarantee that all cohorts have the required number of patients.

## Ethical Considerations

The study will be conducted in compliance with the Declaration of Helsinki, Good Clinical Practice, and national laws and guidelines. The protocol was approved by the London Surrey Research Ethics Committee (REC) NHS Health Research Authority (HRA) and Health and Care Research Wales on 29 June 2023 (REC reference: 23/LO/0492 and IRAS project ID: 312298).

Due to the non‐invasive nature of this study, there is minimal risk involved in using these models for RARP. There is a risk of inaccurately displaying lesions on 3D models, which might affect resection. However, this is highly unlikely since 3D models will be constructed from medical images of the patient. To mitigate this risk, radiologists will confirm the identification of prostate anatomical structures and that cancer lesions accurately represent those on medical images, while surgeons will always use the models in the context of the source MRI.

## Data Protection

Patient privacy is one of the main issues when collecting and sharing data. In this feasibility study we will pseudo‐anonymise patient data to respect patient privacy. Methods for database locking at the start of the analysis and storage upon completion of the clinical investigation will be implemented. Data records will be set to read‐only on completion of data collection for analysis.

## Author Contributions

This study was initiated and designed by A.G. and P.D. A.G. sought ethical approval from the REC HRA. A.G., P.D. and S.O. received funding to execute this study. The manuscript was written by N.K. and A.G., with critical approvals of the final version by N.R, A.S and P.D. Inclusion/exclusion criteria were finalised by F.M., N.R., A.G., D.P. and N.K. D.P. designed the pipeline for prostate anatomy delineation on mpMRI. A.G., M.A. and A.D.‐P. contributed with their expertise in anatomy segmentation for the generation of models. N.B., M.H., C.S. and C.B. contributed with their expertise in 3D printed models. M.C., A.S., A.B., G.G. and F.M. contributed with discussion for a subsequent international multi‐institutional study. E.H. contributed with his experience in 3D virtual models. All authors discussed the results and contributed to the final manuscript.

## Disclosure

Prokar Dasgupta is an advisor to Proximie and Chief Medical Adviser to MysteryVibe. No other named authors in this manuscript have any conflicts of interest to disclose.

## Data Availability

Three‐dimensional printed models will be collected by the Clinical Research Fellow after being used during surgery and then returned to the chief investigator. Models will be disposed of in accordance with hospital disposal policies. There will be no identifying information on the models. The laptop running Innersight Labs 3D visualisation will be returned to the chief investigator after study completion. Data collected will be available on request from corresponding authors.
